# Laparoscopic cholecystectomy for patients with accessory liver lobe attached to the wall of the gallbladder: case reports

**DOI:** 10.1186/s40792-024-01923-9

**Published:** 2024-05-13

**Authors:** Hirotaka Furuke, Tsuyoshi Takagi, Hiroki Kobayashi, Kanehisa Fukumoto

**Affiliations:** 1 Department of Surgery, Nishijin Hospital, 1035 Mizomae-cho, Kamigyo-ku, Kyoto, 6028319 Japan; 2 Division of Digestive Surgery, Department of Surgery, Kyoto Prefectural University of Medicine, Kyoto, Japan

**Keywords:** Laparoscopic cholecystectomy, Accessory liver lobe, Postoperative complications

## Abstract

**Background:**

Laparoscopic cholecystectomy (LC) is one of the most commonly undertaken procedures worldwide for cholecystolithiasis and cholecystitis. Accessory liver lobe (ALL) is a developmental anomaly defined as an excessive liver lobe composed of a normal liver parenchyma. Some ALL exist on the serosal side of the gallbladder. We herein present two cases of ALL incidentally detected during LC.

**Case presentation:**

The first case was a 69-year-old woman diagnosed with chronic cholecystitis. LC was performed. ALL was observed anterior to the wall of the gallbladder and resected after clipping. Pathological findings revealed liver tissue with Glisson’s capsule and a lobular structure in ALL. However, communication between the bile ducts of ALL and the main liver was unclear due to surgical heat degeneration. The second case was a 56-year-old woman diagnosed with acute cholecystitis. LC was performed approximately one month after the attack, and ALL attached to the wall of gallbladder. ALL was clipped and completely resected. Pathological findings showed that the bile ducts of ALL might be connected within the wall of gallbladder.

**Conclusions:**

We presented two cases of ALL attached to the gallbladder encountered during LC. Since ALL contains a normal liver parenchyma, postoperative bleeding or bile leakage may occur if it is inefficiently resected. Therefore, the complete resection of ALL is important to prevent these postoperative complications.

## Background

The prevalence of gallstones is as high as 10–15% in the global adult population [1]. Cholecystolithiasis or cholecystitis occurs in approximately 20% of patients with gallstones, and cholecystectomy is recommended [2, 3]. Laparoscopic cholecystectomy (LC) is one of the most commonly undertaken procedures worldwide [4]. Bile leakage or bleeding are well-known complications of LC, and management is required for their prevention [5].

Accessory liver lobe (ALL) is a developmental anomaly defined as an excessive liver lobe composed of a normal liver parenchyma [6, 7]. Since most cases of ALL are asymptomatic, treatment is not necessary. However, in rare cases, pedunculated ALL occur on the serosal side of the gallbladder [8–10]. Surgeons may encounter ALL attached to the gallbladder during LC. Appropriate management for ALL is necessary to avoid postoperative bleeding or bile leakage. We herein present two cases of ALL encountered during LC with a literature review.

## Case presentation

**Fig. 1:**
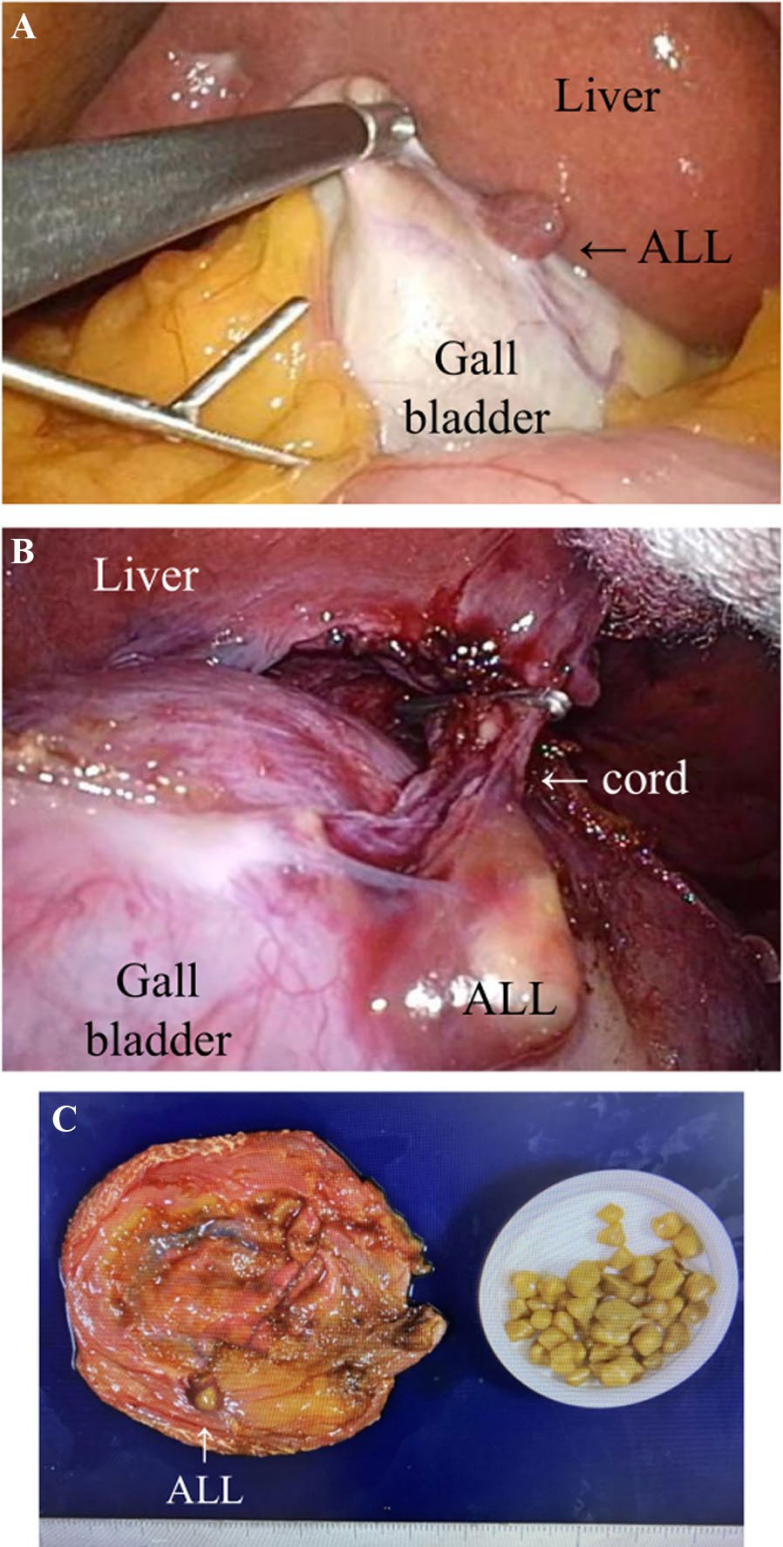
Intraoperative findings of the first case. **A** ALL was observed at the anterior peritoneal side of the gallbladder. The location was on the fundus side. The size of ALL was approximately 7 mm. **B** The cord-like substance between the main liver and ALL was resected after clipping. **C** A photo of the resected specimen. ALL was completely resected. Cholesterol gallstones were present in the gallbladder. ALL: accessory liver lobe

**Fig. 2:**
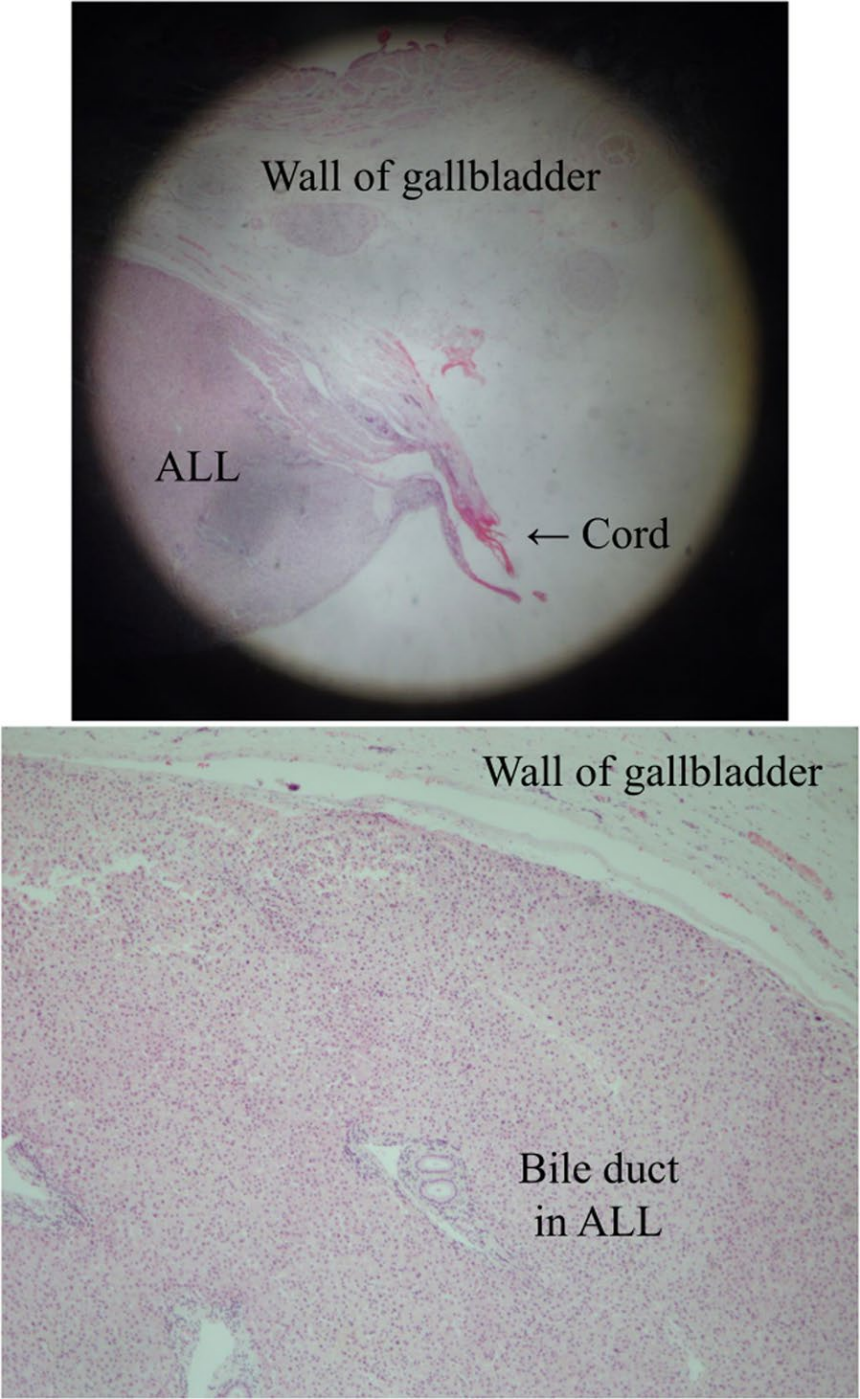
Hematoxylin–eosin staining of ALL, the wall of gallbladder, and cord-like substance (at × 20 and × 200 magnification, respectively). Liver tissue with Glisson’s capsule and a lobular structure were observed in ALL. Bile ducts were confirmed in ALL; however, communication with the main liver was unclear due to the heat denaturation of the tissue. ALL: accessory liver lobe

The first case was a 69-year-old woman diagnosed with chronic cholecystitis. She had a previous medical history of appendectomy for acute appendicitis at 11 years and hysterectomy for uterine fibroids at 32 years. ALL was not detected by computed tomography (CT) or magnetic resonance imaging (MRI) before surgery. LC was performed for chronic cholecystitis at our institution. The port was inserted into the umbilicus and the gallbladder was observed (we performed LC using a single port). Inflammatory adhesion of the omentum was noted around the gallbladder. After its detachment, ALL was observed at the anterior peritoneal side of the gallbladder. The location was on the fundus side. The size of ALL was approximately 7 mm, and the cord-like substance between the main liver and ALL was resected after clipping. Cholecystectomy was routinely performed. The surgical view and extracted gallbladder and gallstones are shown in Fig. 1. There were no postoperative complications, and the patient was discharged on the third day after surgery. Pathological findings revealed cholecystolithiasis with cholesterosis and Rokitansky–Aschoff sinuses. Liver tissue with Glisson’s capsule and a lobular structure were observed in ALL. Bile ducts were confirmed in ALL; however, their communication with the main liver was unclear due to surgical heat degeneration (Fig. 2).

**Fig. 3:**
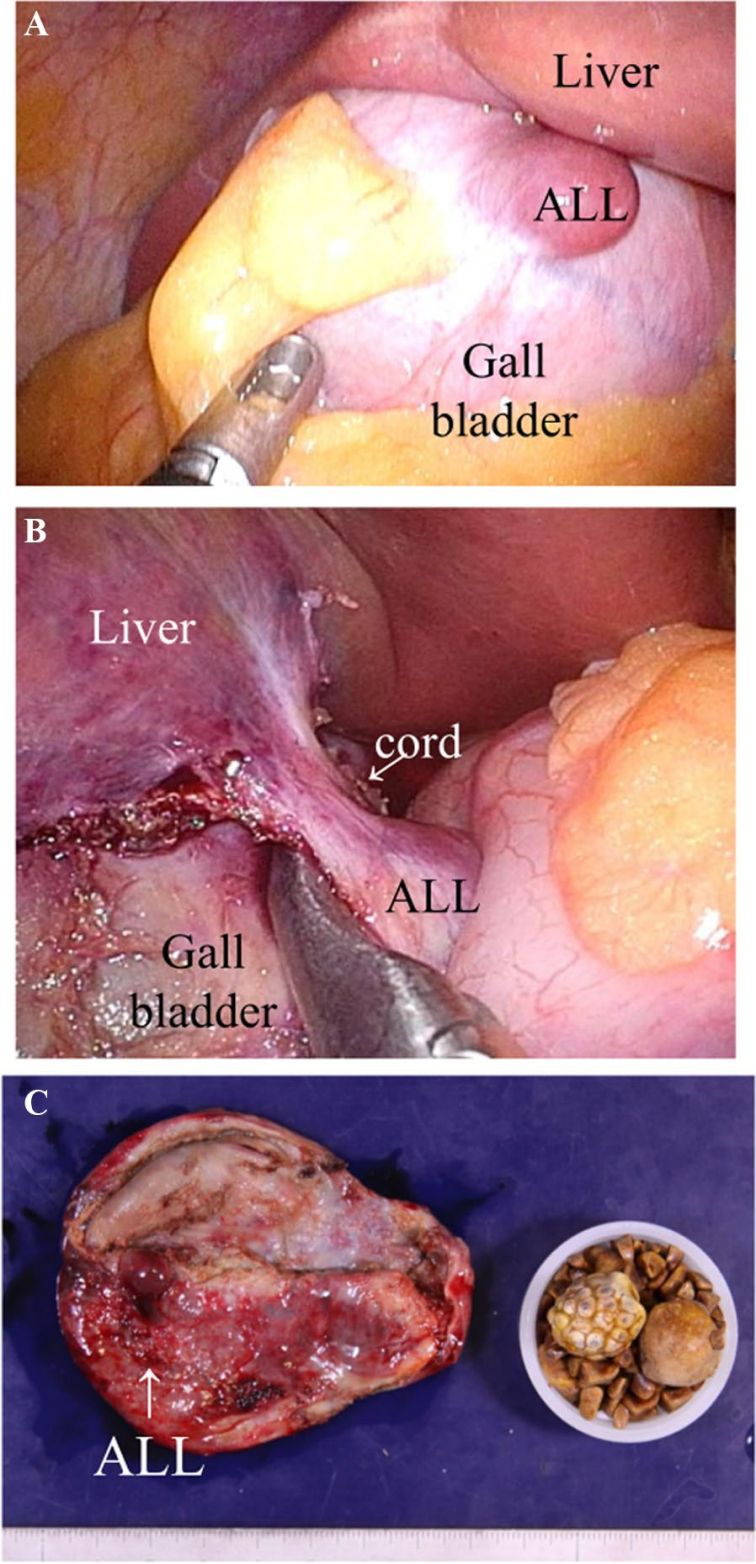
Intraoperative findings of the second case. **A** Similar to the first case, ALL was observed at the peritoneal side of the gallbladder. The location was on the anterior, and fundus side. The size of ALL was approximately 10 mm. **B** The cord-like substance between the main liver and ALL was clipped and resected. **C** A photo of the resected specimen. ALL was on the serosal surface of the gallbladder. ALL: accessory liver lobe

**Fig. 4:**
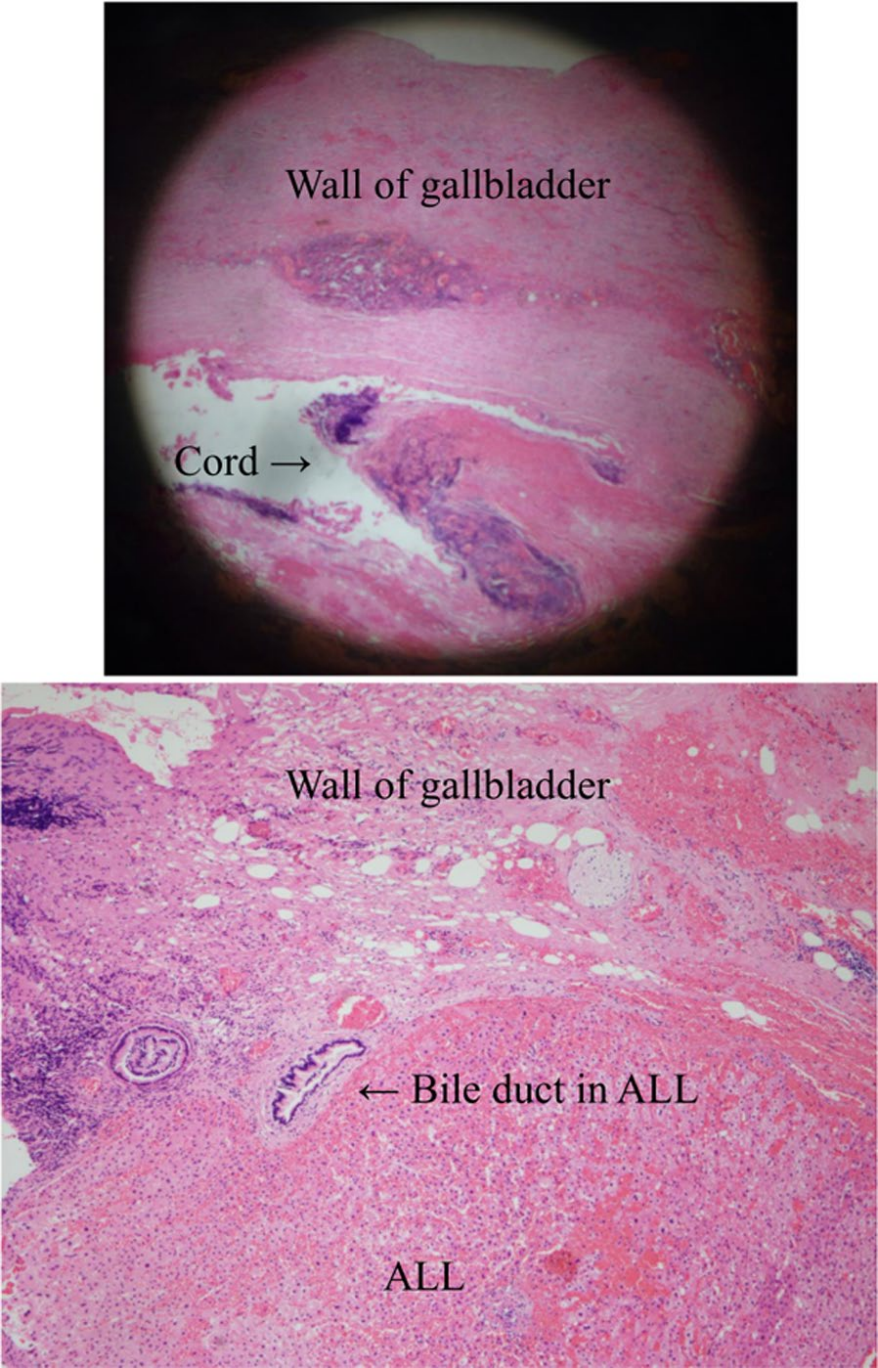
Hematoxylin–eosin staining of ALL, the wall of gallbladder, and cord-like substance (at × 20 and × 200 magnification, respectively). ALL consisted of liver tissue with Glisson’s capsule and a lobular structure. Bile ducts in ALL were connected within the gallbladder wall, suggested the bile ducts in ALL opened into gallbladder. However, the connection between the main liver was unclear due to the heat denaturation of the tissue. ALL: accessory liver lobe

The second case was a 56-year-old woman diagnosed with acute cholecystitis. Acute cholecystitis improved with antibiotic treatment, and the patient was referred for elective cholecystectomy. She had a previous history of surgical treatment for an ovarian cyst and uterine fibroids. Similar to the first case, ALL was not detected by CT or MRI before surgery. LC was performed approximately one month after the attack. Inflammation of the gallbladder was severe and wall thickening was observed. ALL was observed at the peritoneal side of the gallbladder. The location was on the anterior, and fundus side. Its size was 10 mm (Fig. 3). The cord-like substance between the main liver and ALL was clipped and resected (Fig. 3). Cholecystectomy was performed stereotypically. There were no complications, and the patient was discharged on the second day after surgery. Pathological findings revealed cholecystolithiasis with fibrosis. Bile ducts in ALL were connected within the gallbladder wall, suggested the bile ducts in ALL opened into gallbladder (Fig. 4).

## Discussion

ALL is defined as a supernumerary liver lobe composed of a normal liver parenchyma, including bile ducts and blood vessels [6]. Previous studies reported that ALL was caused by embryological abnormalities [11] or acquired trauma [12]. There are three types of ALL. Riedel’s lobe is a tongue-like elongation of the right liver, an ectopic lobe is completely independent of the liver parenchyma, which may exist anywhere in the body, and a pedunculated lobe is ALL that is continuous with the liver [6, 13]. The prevalence of Riedel’s lobe ranges between 3.3 and14.5% [14, 15] and is more frequent in women (4.5–19.4%) than in men (2.1–6.1%) [6]. Most cases of ALL are asymptomatic; however, torsion [16], bleeding [17], or extrinsic compression of the stomach [18] have been reported. A previous study showed that the incidence of ALL (pedunculated lobe) was 0.7% [10]. We herein presented two cases of ALL attached to the gallbladder during LC. ALL was classified as pedunculated lobes in both of these cases.

The diagnosis of ALL during preoperative imaging examinations may facilitate its treatment in surgery. Ultrasonography, enhanced CT, and MRI may be useful for its diagnosis before surgery [19]. However, in the two present cases, ALL was not detected in preoperative examinations. The size of ALL attached to the gallbladder was approximately 10 mm and it was compressed by the liver in a supine position; therefore, it was difficult to diagnose before surgery. Since ALL may be incidentally encountered, it is necessary to always be prepared for its treatment.

Pathological findings previously demonstrated that ALL contains three major structures: a portal vein, hepatic artery, and bile ducts [8, 9]. Vessels and bile ducts in ALL (particularly pedunculated lobes) may be connected to the main liver. Therefore, postoperative bleeding or bile leakage may occur if ALL is not completely resected. It is desirable to clip and resect bile ducts that enter the gallbladder bed (for example, a subvesical bile duct) in order to avoid postoperative bile leakage [20]. We resected the cord between ALL and the main liver using laparoscopic coagulation shears after clipping, and both cases were discharged without bile leakage after surgery. Due to surgical heat degeneration of the cord, communication between bile ducts in ALL and the main liver was unclear. Instead, bile ducts in ALL might be opened into the gallbladder in the second case; therefore, the preservation of ALL may also be a cause of bile leakage. It was very rare anatomy if the bile ducts in ALL opening directly into the gallbladder. However, there were bile ducts that communicate with the gallbladder other than cystic duct, such as Luschka's ducts or subvesical bile ducts [20]. It might be possible that the bile duct in ALL opening into the gallbladder due to an embryological abnormality. For our suggestion, ALL attached to the gallbladder needs to be completely resected along with the gallbladder in order to avoid bile leakage.

Since this was a retrospective observational study at a single institution with a small number of patients, further research on a larger number of patients is necessary.

## Conclusion

We herein presented two cases of ALL attached to the gallbladder that were encountered during LC. It is important to always consider ALL attached to the wall of the gallbladder when performing LC. To avoid postoperative complications, ALL, particularly pedunculated lobes, need to be completely resected after clipping the cord-like substance.

## Data Availability

The data for this study will not be shared to ensure patient confidentiality.

## Abbreviations

LC: Laparoscopic cholecystectomy
ALL: Accessory liver lobe
CT: Computed tomography
MRI: Magnetic resonance imaging
